# Orchestrated multi agents sustain accuracy under clinical-scale workloads compared to a single agent

**DOI:** 10.1038/s44401-026-00077-0

**Published:** 2026-03-09

**Authors:** Eyal Klang, Mahmud Omar, Ganesh Raut, Reem Agbareia, Prem Timsina, Robert Freeman, Nicholas Gavin, Lisa Stump, Alexander W. Charney, Benjamin S. Glicksberg, Girish N. Nadkarni

**Affiliations:** 1 The Windreich Department of Artificial Intelligence and Human Health, Mount Sinai Medical Center, New York, NY USA; 2 The Division of Data-Driven and Digital Medicine (D3M), Icahn School of Medicine at Mount Sinai, New York, NY USA; 3 The Hasso Plattner Institute of Digital Health, Icahn School of Medicine at Mount Sinai, New York, NY USA; 4 Ophthalmology Department, Hadassah Medical Center, Jerusalem, Israel; 5 Mindich Child Health and Development Institute and the Departments of Pediatrics and Genetics & Genomic Sciences, Icahn School of Medicine at Mount Sinai, New York, NY USA

**Keywords:** Engineering, Mathematics and computing

## Abstract

We tested state-of-the-art LLMs under clinical-scale workloads using two designs: a single agent handling all tasks and a multi-agent orchestrator assigning each task to a dedicated worker. Across retrieval, extraction, and dosing tasks, batch sizes ranged from 5–80. Multi-agent accuracy remained high (90.6% at 5 tasks; 65.3% at 80), while single-agent accuracy collapsed (73.1% to 16.6%; *p* < 0.01). Multi-agent runs used up to 65-fold fewer tokens and limited latency growth. These findings show that lightweight orchestration preserves accuracy and efficiency under mixed-task clinical loads.

Large-language models (LLMs) are already used in clinical research and practice because they can read free text, reason over symptoms and generate fluent advice^1^. Recent work wraps these models in autonomous “agents” that decide, for example, which database query or dose calculator to invoke at each step, reflecting the multi-step structure of clinical problem-solving^2^. AMIE, published in Nature, showed that a single dialogue‑optimised LLM can match physicians in simulated consultations^3^. DRAGON introduced a multi‑task clinical NLP benchmark with per‑task evaluation^4^, and Liu et al. documented long‑context degradation, including the “lost‑in‑the‑middle” effect^5^. These studies assess one case or one task at a time and do not vary concurrent batch size, task heterogeneity, or tool diversity. We test how mixed clinical batches may amplify these effects and whether lightweight orchestration preserves accuracy and cost under load.

We conduct a study that tests state-of-the-art LLM agents in two execution modes to examine scalability under mixed-task clinical workloads. In the single-agent baseline one agent receives an entire batch of heterogeneous tasks. In the orchestrated mode with multiple agents a lightweight orchestrator sends each task to a dedicated worker agent, which calls one domain tool before returning its answer for aggregation. Batch sizes ranged from 5 to 80 tasks to mimic clinical traffic. Tasks fell into three categories: Retrieval (find PubMed abstracts), Extraction (pull fields from a discharge note), and Dosing (solve a medication‑math calculation) (tasks are detailed in Supplement Section 1). These tasks are not intended to represent the full scope of clinical work such as free-form summarization or medical reasoning. Rather, they serve as common workflow primitives- retrieve evidence, extract key fields, and compute deterministic values- that are often embedded inside broader end-to-end clinical applications. This let us test whether task‑level delegation preserves accuracy and limits latency and token cost as conversational agents scale, while keeping every reasoning step transparent and traceable (Fig. 1).

**Fig. 1:**
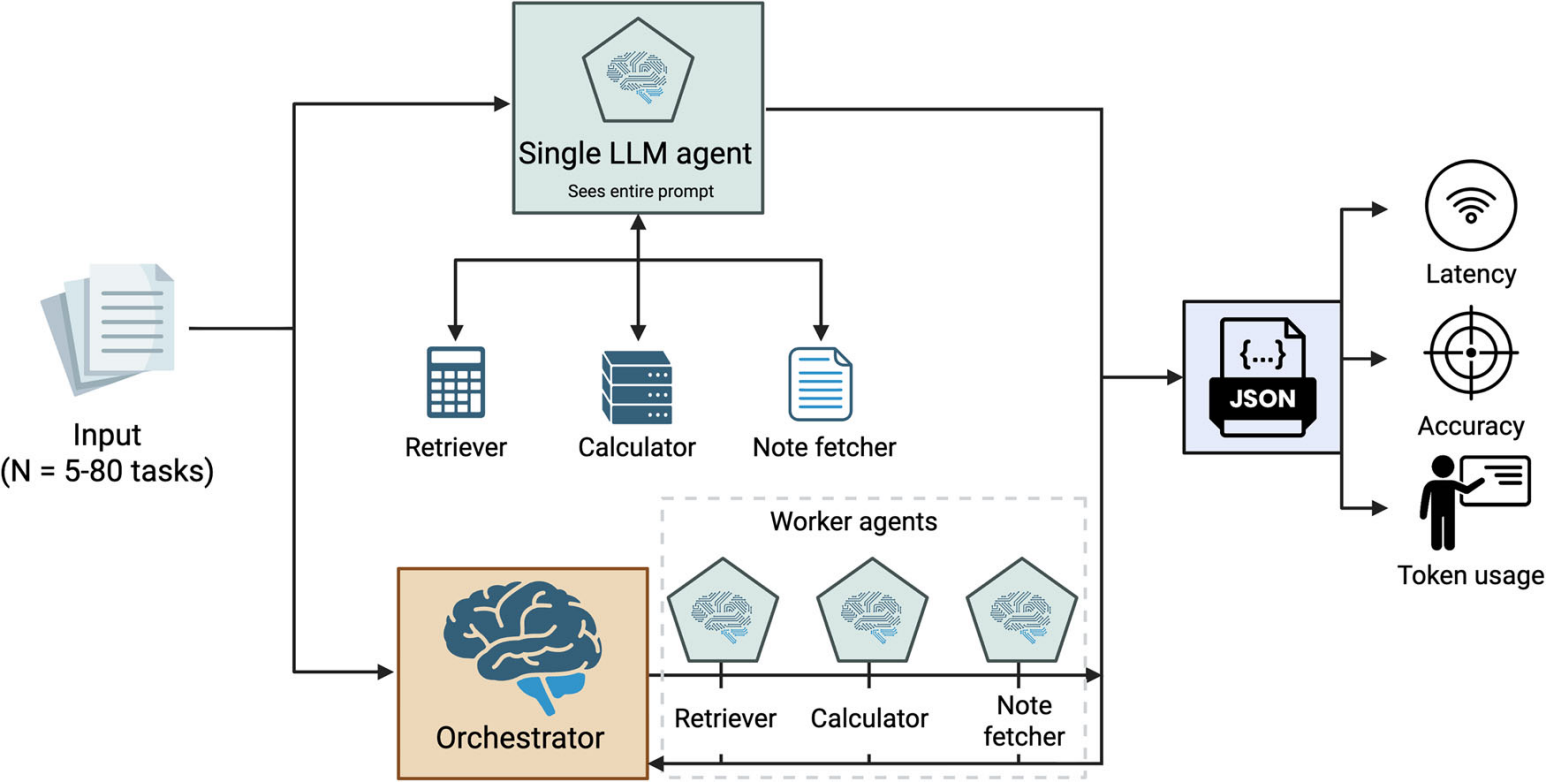
Overview of the pipeline design.

In our tests, accuracy diverged once batch size passed ten tasks (Fig. 2). When results were pooled, multi‑agent runs held 90.6% at batches of five tasks and 65.3% at eighty (95% CI 56–74%), whereas single‑agent accuracy fell from 73.1% to 16.6%. GPT‑4.1‑mini illustrates the gap: its multi‑agent accuracy stayed between 96% and 91.4% across all batch sizes, while the single agent declined from 96% to 33.9%. GPT‑4.1‑nano, Llama‑2‑70B and Qwen‑3‑8B showed the same pattern, with single‑agent accuracy dropping far below their multi‑agent counterparts. All differences between topologies beyond ten tasks were significant (Welch t‑test, FDR‑adjusted *p* < 0.01). Detailed values are listed in Supplementary Section 1.

**Fig. 2:**
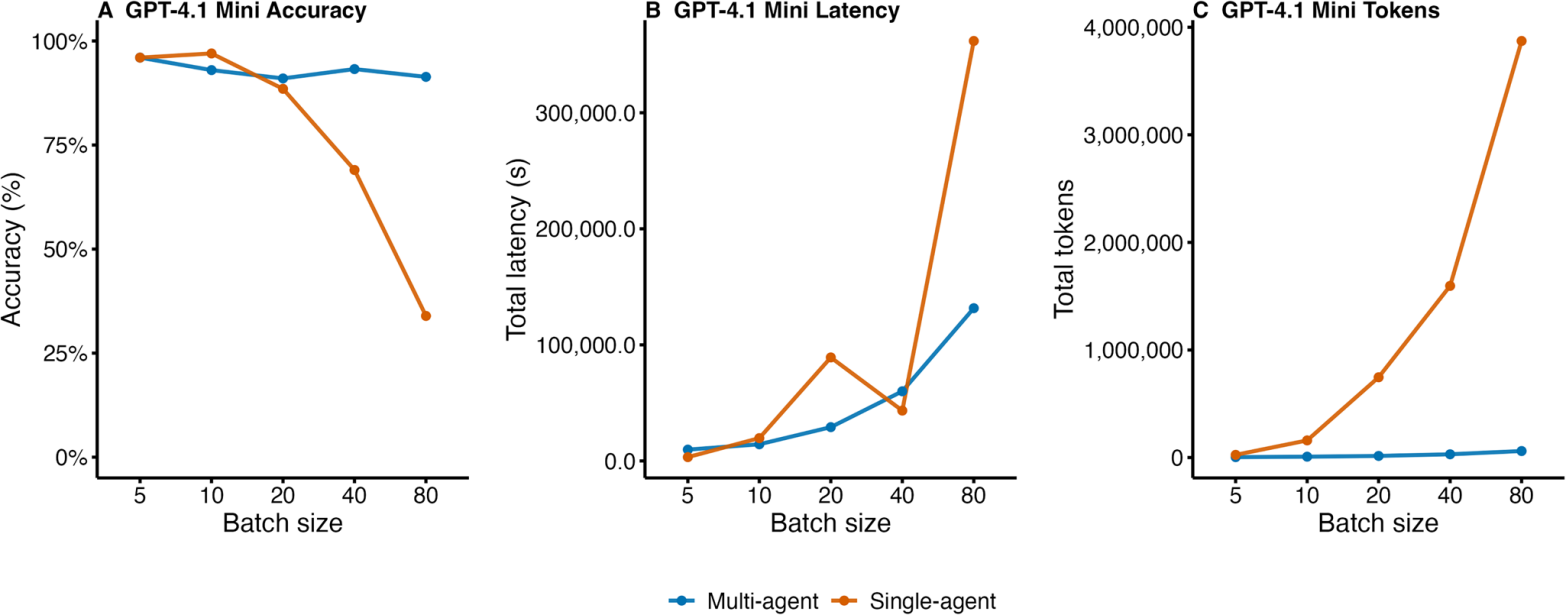
Accuracy and token usage. **A** Accuracy of GPT-4.1 across batch sizes, **B** latecny of the same pipeline across batch sizes and **C** total token usage across batch sizes.

Token growth differed sharply (Supplementary Figure S2 and Table S3) between agent types. For GPT‑4.1‑mini the multi‑agent batch rose only from 3.7 k tokens at five tasks to 60 k at eighty, whereas the single agent expanded from 25 k to 3.9 M—a 65‑fold gap at the highest load. GPT‑4.1‑nano showed the same direction but at higher totals. Llama‑2‑70B and Qwen‑3‑8B were less consistent; many large single‑agent batches for these models failed early and returned no JSON, confounding direct comparison.

Total latency rose with batch size in both modes (Supplementary Figure S3 and Table S2**)**, but the single‑agent curve climbed faster. For GPT‑4.1‑mini, multi‑agent batches grew from 9 728 s at five tasks to 131 603 s at eighty, whereas single‑agent batches rose from 3 258 s to 361 864 s. The other models showed the same direction: multi‑agent latency increased predictably, while single‑agent runs jumped higher and displayed wider confidence intervals; several large single‑agent batches for Qwen‑3‑8B and Llama‑2‑70B failed and returned no JSON.

Delegating each task to its own worker appears to insulate the LLM from context interference, so accuracy remains high even when many unrelated prompts arrive at once. This insulation likely stems from two linked mechanisms. First, each worker receives only the tokens that matter for a single decision, so attention is not diluted across irrelevant material^6^. Second, the orchestrator re-assembles answers without expanding the prompt seen by any one model call, keeping the effective context well within the range the model was tuned to handle^7^. Orchestration introduces a fixed overhead (orchestrator + worker calls and repeated prompts), which can increase total tokens and latency at smaller batch sizes for some checkpoints; however, as batch size grows, task partitioning prevents context-driven degradation and yields more predictable scaling under load.

Model scale in this pipeline appears to drive stability. The larger the checkpoint, the longer accuracy persists under multi-agent load. GPT-mini, likely the most parameter-rich model in the ensemble, is by far the best performer and barely drifts from its initial high accuracy. GPT-nano, consistent with its smaller size, begins to erode once batch size rises. Llama-2-70B follows the same pattern and sheds more accuracy as batches grow. As expected from this trend, Qwen-3-8B, with the smallest parameter budget and limited exposure to mixed-tool prompts, degrades fastest.

This is the first study to show that a lightweight orchestrator can turn existing LLMs into reliable, token-efficient teams for complex clinical workflows. By dividing labour into single-tool subtasks, the architecture keeps accuracy high even when many requests arrive simultaneously- an essential property for automated, large-scale use. Recent agent studies in medicine, AMIE for diagnostic dialogue^3^, DRAGON for radiology reporting^4^, and a handful of retrieval-augmented chart summarizers, each examined one model, one patient at a time, and did not examine performance, cost or latency under clinical scale loads^8^. Our experiments address a different but linked and practical aspect: context overload when dozens of heterogeneous tasks arrive together. The findings show that an orchestrator-worker layout sustains accuracy, tokens, and latency in a regime where single-prompt agents collapse. This benefit holds across four checkpoints with widely different sizes and pre-training recipes, suggesting the gain comes from task partitioning rather than from any model-specific optimisation. By constraining each worker to a single tool and a short prompt, the design also produces a transparent audit trail, as every intermediate call can be logged and replayed, addressing a key regulatory concern that earlier end-to-end agents leave unresolved.

This study uses curated retrieval and extraction sets, which allow controlled batch evaluation but do not replace testing on real EHR data; future work may assess the same orchestration approach directly on live clinical notes. Our evaluation focuses on pipeline scalability using tasks with deterministic ground truth, rather than on clinical decision-making. Applying this orchestration approach to real clinical workflows will require further validation, including clinician review, which we plan to explore in future work. We also required structured JSON outputs for automated evaluation; future work should assess whether the same scalability trends hold under free-form (plain-text) outputs that may reduce overhead for some models

Our multi-agent approach therefore offers a practical and auditable way to scale agentic execution for mixed-task pipelines under increasing load. Future studies should test similar orchestration on end-to-end applications (including summarization and medical reasoning) and on real clinical notes, with appropriate clinician review, to assess external validity.

## Methods

### Experimental protocol and agentic pipelines

We tested two agent layouts on three task classes: retrieving oncology abstracts, extracting fields from discharge notes and solving numeric dosing problems. The retrieval set is a FAISS index of 234,650 PubMed records on neoplasms (2020-01-01 to 2025-05-30) embedded with GIST-large. The extraction set contains 331,793 electronic health record (EHR) summaries with admission and discharge dates jittered across 2005-2019^9^. The calculation set is generated by 20 templates that cover routine weight-, surface- and clearance-based dosing. Ground truth is a single canonical string per item; accuracy is the proportion of exact matches after case- and whitespace normalisation (More detailed description is provided in the Supplementary Sections 2 and 3). Evaluation was fully automated (exact-match after normalization); no manual scoring was used in the reported experiments. The full dataset construction and evaluation pipeline—data sources, inclusion rules, task prompts, target/label definitions, normalization, and scoring—is detailed in Supplement Sections 2–3. In brief, each task instance is generated from its source corpus or template, paired with a deterministic target output, executed under the specified agent topology, and scored via exact-match after normalization.

In this study, an orchestrator is a controller that receives a list of tasks, selects the relevant tool for each task, and dispatches the task to a worker. A worker agent is a model instance configured to handle one task at a time using one specific tool. In the single-agent condition, one model receives the full batch and may call all tools; in the multi-agent condition, the orchestrator routes each task to one worker that uses only its assigned tool.

Agents could call a k-NN retriever, a sandboxed calculator and a note fetcher. In the single-agent condition one language model received the full batch of *N* tasks and up to 10 × *N* turns. In the multi-agent condition an orchestrator dispatched each task to one worker, each worker handled one tool, and the orchestrator merged the replies. Four instruction-tuned checkpoints ran under both topologies with random seed 42.

Batch sizes were 5, 10, 20, 40 and 80; ten non-overlapping batches were generated for every model-topology pair. Open-weight models used an 8 × H100-80 GB node; hosted models were called through vendor APIs. We recorded wall-clock latency from the first call to receipt of a *valid final JSON* response and counted total input + output tokens across all calls. Both topologies were evaluated under the same machine-readable JSON output requirement to enable automated scoring and consistent batching; this constraint is an evaluation harness choice and does not imply that clinical user-facing outputs must be JSON. Representative examples are provided in Supplementary Section 4.

### Statistical analysis

Accuracy was evaluated with two-sided Welch t-tests; latency and token totals with Wilcoxon rank-sum tests. *P* values were Benjamini–Hochberg corrected within each metric family. Analyses ran in R 4.3 and full code and logs are supplied in the Supplementary Section 3.

### Ethical approval

This work was conducted under an institutional review board approval.

## Supplementary information

Supplementary Information

## Data Availability

All data generated in the reported study can be provided to research requests from other individuals or institutions upon a reasonable request from the corresponding author within a month.
