# Supplementary material for: Orchestrated multi agents sustain accuracy under clinical-scale workloads compared to a single agent
Source: Npj Health Syst. 2026 Mar 9;3:23. doi: 10.1038/s44401-026-00077-0 (PMC13354228; doi:10.1038/s44401-026-00077-0)
Supplement: Supplementary file 1 — Supplementary Information [file 44401_2026_77_MOESM1_ESM.pdf]

Section 1: full results.

Column definitions for the following tables:

Column	Meaning
dataset	Model checkpoint evaluated (for example, *gpt_mini* or *llama-2-70B*).
type	Agent topology: *multi* = multi-agent with orchestrator; *single* = single-agent baseline.
N_tasks	Batch size, that is, number of tasks processed together.
n	Number of independent batches run for that condition (replicates).
mean_acc	Mean accuracy across the *n* batches (exact-match rate).
se_acc	Standard error of the mean accuracy.
ci_low, ci_high	Lower and upper bounds of the two-sided 95 % confidence interval for the mean accuracy.

Table S1. Accuracy by dataset × agent × batch size.

dataset	type	N_tasks	n	mean_acc	se_acc	ci_low	ci_high
**gpt_mini**	multi	5	10	96.0%	2.7%	90.0%	100.0%
**gpt_mini**	multi	10	10	93.0%	2.1%	88.2%	97.8%
**gpt_mini**	multi	20	10	91.0%	2.8%	84.7%	97.3%
**gpt_mini**	multi	40	10	93.3%	0.7%	91.6%	94.9%
**gpt_mini**	multi	80	10	91.4%	1.7%	87.5%	95.3%
**gpt_mini**	single	5	10	96.0%	2.7%	90.0%	102.0%
**gpt_mini**	single	10	10	97.0%	1.5%	93.5%	100.5%
**gpt_mini**	single	20	10	88.5%	2.6%	82.6%	94.4%
**gpt_mini**	single	40	10	69.0%	6.7%	53.9%	84.1%
**gpt_mini**	single	80	10	33.9%	2.8%	27.6%	40.2%
**gpt_nano**	multi	5	10	82.0%	6.3%	67.8%	96.2%
**gpt_nano**	multi	10	10	73.3%	7.9%	55.4%	91.2%
**gpt_nano**	multi	20	10	71.9%	4.3%	62.1%	81.6%
**gpt_nano**	multi	40	10	71.8%	3.8%	63.1%	80.4%
**gpt_nano**	multi	80	10	59.3%	5.2%	47.4%	71.1%
**gpt_nano**	single	5	10	60.0%	4.5%	49.9%	70.1%
**gpt_nano**	single	10	10	42.2%	6.1%	28.5%	56.0%
**gpt_nano**	single	20	10	33.8%	4.5%	23.5%	44.0%
**gpt_nano**	single	40	10	16.3%	2.0%	11.6%	20.9%
**gpt_nano**	single	80	10	10.4%	2.7%	4.1%	16.6%
**llama_xlam_2_70b**	multi	5	10	92.0%	3.3%	84.6%	99.4%
**llama_xlam_2_70b**	multi	10	10	93.0%	1.5%	89.5%	96.5%
**llama_xlam_2_70b**	multi	20	10	78.9%	6.5%	64.3%	93.5%
**llama_xlam_2_70b**	multi	40	10	76.9%	6.2%	63.0%	90.9%

**llama_xlam_2_70b**	multi	80	10	83.6%	3.3%	76.1%	91.0%
**llama_xlam_2_70b**	single	5	10	90.0%	3.3%	82.5%	97.5%
**llama_xlam_2_70b**	single	10	10	91.1%	1.9%	86.8%	95.4%
**llama_xlam_2_70b**	single	20	10	47.5%	6.4%	33.1%	61.9%
**llama_xlam_2_70b**	single	40	10	32.1%	6.8%	16.7%	47.4%
**qwen_3_8B**	multi	5	10	100.0%	0.0%	100.0%	100.0%
**qwen_3_8B**	multi	10	10	91.0%	1.8%	86.9%	95.1%
**qwen_3_8B**	multi	20	10	81.5%	2.9%	75.0%	88.0%
**qwen_3_8B**	multi	40	10	69.7%	7.2%	53.5%	85.9%
**qwen_3_8B**	multi	80	10	29.0%	8.1%	10.6%	47.4%
**qwen_3_8B**	single	5	10	25.7%	4.8%	14.9%	36.5%
**qwen_3_8B**	single	10	10	6.7%	1.8%	2.5%	10.8%
**qwen_3_8B**	single	20	10	3.0%	0.9%	1.0%	5.0%
**qwen_3_8B**	single	40	10	1.9%	0.4%	1.0%	2.8%
**qwen_3_8B**	single	80	10	1.3%	0.3%	0.6%	1.9%

Tables S2. Total latency (s) by dataset × agent × batch size.

dataset	type	N_tasks	n	mean_val	se_val	ci_low
**gpt_mini**	multi	5	10	9,728.00	2,335.08	4,445.69
**gpt_mini**	multi	10	10	14,333.50	110.49	14,083.56
**gpt_mini**	multi	20	10	29,098.80	389.74	28,217.14
**gpt_mini**	multi	40	10	60,002.90	327.57	59,261.88
**gpt_mini**	multi	80	10	131,603.00	2,344.26	126,299.91
**gpt_mini**	single	5	10	3,258.40	448.82	2,243.10
**gpt_mini**	single	10	10	19,641.50	6,352.34	5,271.51
**gpt_mini**	single	20	10	89,159.50	22,890.47	37,377.66
**gpt_mini**	single	40	10	43,344.50	12,411.57	15,267.57
**gpt_mini**	single	80	10	361,863.70	179,003.44	-43,070.21
**gpt_nano**	multi	5	10	6,414.50	58.66	6,281.80
**gpt_nano**	multi	10	10	13,251.70	513.14	12,090.90
**gpt_nano**	multi	20	10	28,573.70	1,955.27	24,150.58
**gpt_nano**	multi	40	10	51,440.80	1,511.29	48,022.03
**gpt_nano**	multi	80	10	100,550.70	5,647.78	87,774.53
**gpt_nano**	single	5	10	5,384.70	609.12	4,006.78
**gpt_nano**	single	10	10	7,845.30	659.17	6,354.16
**gpt_nano**	single	20	10	11,743.00	1,781.59	7,712.76
**gpt_nano**	single	40	10	25,231.30	8,822.03	5,274.48
**gpt_nano**	single	80	10	14,181.50	4,619.84	3,730.70
**llama_xlam_2_70b**	multi	5	10	85,567.30	3,708.85	77,177.29
**llama_xlam_2_70b**	multi	10	10	194,376.00	3,785.55	185,812.49
**llama_xlam_2_70b**	multi	20	10	454,734.90	39,026.26	366,451.36

**llama_xlam_2_70b**	multi	40	10	838,632.60	98,746.13	615,253.33
**llama_xlam_2_70b**	multi	80	10	3,315,372.10	1,510,773.36	-102,234.68
**llama_xlam_2_70b**	single	5	10	57,707.30	4,482.54	47,567.10
**llama_xlam_2_70b**	single	10	10	179,635.30	11,180.75	154,342.69
**llama_xlam_2_70b**	single	20	10	312,827.90	76,878.36	138,916.97
**llama_xlam_2_70b**	single	40	10	578,546.30	113,230.47	322,401.18
**qwen_3_8B**	multi	5	10	321,068.90	47,051.26	214,631.55
**qwen_3_8B**	multi	10	10	272,956.10	123,639.05	-6,734.85
**qwen_3_8B**	multi	20	10	369,713.40	126,273.13	84,063.75
**qwen_3_8B**	multi	40	10	1,046,207.90	621,769.29	-360,331.95
**qwen_3_8B**	multi	80	10	2,036,080.70	847,342.92	119,257.85
**qwen_3_8B**	single	5	10	3,789.10	414.59	2,851.23
**qwen_3_8B**	single	10	10	3,070.20	412.86	2,136.25
**qwen_3_8B**	single	20	10	2,835.20	290.39	2,178.28
**qwen_3_8B**	single	40	10	2,722.60	310.32	2,020.60
**qwen_3_8B**	single	80	10	285,249.40	192,619.99	-150,487.30

Table S3. Total tokens by dataset × agent × batch size.

dataset	type	N_tasks	n	mean_val	se_val	ci_low	ci_high
**gpt_mini**	multi	5	10	3,744	226	3,231	4,256
**gpt_mini**	multi	10	10	7,935	244	7,383	8,486
**gpt_mini**	multi	20	10	14,875	217	14,384	15,366
**gpt_mini**	multi	40	10	30,330	382	29,465	31,195
**gpt_mini**	multi	80	10	60,371	275	59,750	60,992
**gpt_mini**	single	5	10	24,751	5,249	12,876	36,626
**gpt_mini**	single	10	10	159,246	32,061	86,720	231,772
**gpt_mini**	single	20	10	746,086	101,337	516,845	975,328
**gpt_mini**	single	40	10	1,595,768	360,754	779,686	2,411,850
**gpt_mini**	single	80	10	3,872,443	670,732	2,355,141	5,389,744
**gpt_nano**	multi	5	10	3,748	226	3,237	4,259
**gpt_nano**	multi	10	10	7,252	783	5,482	9,023
**gpt_nano**	multi	20	10	14,977	271	14,365	15,590
**gpt_nano**	multi	40	10	29,245	1,015	26,949	31,540
**gpt_nano**	multi	80	10	53,209	2,797	46,881	59,536
**gpt_nano**	single	5	10	14,652	1,415	11,450	17,854
**gpt_nano**	single	10	10	68,096	8,975	47,793	88,398
**gpt_nano**	single	20	10	113,612	29,759	46,291	180,932
**gpt_nano**	single	40	10	519,614	156,899	164,682	874,545
**gpt_nano**	single	80	10	891,986	512,762	-267,962	2,051,934

**llama_xlam_2_70b**	multi	5	10	31,032	1,331	28,021	34,043
**llama_xlam_2_70b**	multi	10	10	70,094	2,265	64,970	75,217
**llama_xlam_2_70b**	multi	20	10	199,721	39,169	111,114	288,328
**llama_xlam_2_70b**	multi	40	10	370,747	54,371	247,751	493,744
**llama_xlam_2_70b**	multi	80	10	3,638,240	2,716,54 1	- 2,507,002	9,783,481
**llama_xlam_2_70b**	single	5	10	45,955	7,222	29,617	62,292
**llama_xlam_2_70b**	single	10	10	150,911	15,380	116,120	185,703
**llama_xlam_2_70b**	single	20	10	311,665	92,950	101,397	521,933
**llama_xlam_2_70b**	single	40	10	542,206	131,440	244,868	839,545
**qwen_3_8B**	multi	5	10	351,183	53,239	230,748	471,618
**qwen_3_8B**	multi	10	10	272,039	135,146	-33,682	577,760
**qwen_3_8B**	multi	20	10	329,081	116,630	65,245	592,917
**qwen_3_8B**	multi	40	10	917,252	462,153	-128,210	1,962,714
**qwen_3_8B**	multi	80	10	1,691,343	607,848	316,296	3,066,390
**qwen_3_8B**	single	5	10	3,898	477	2,819	4,976
**qwen_3_8B**	single	10	10	4,740	623	3,332	6,149
**qwen_3_8B**	single	20	10	4,516	250	3,949	5,083
**qwen_3_8B**	single	40	10	6,486	456	5,453	7,518
**qwen_3_8B**	single	80	10	161,092	104,749	-75,866	398,050

Supplementary figures for the single model results.

Figure

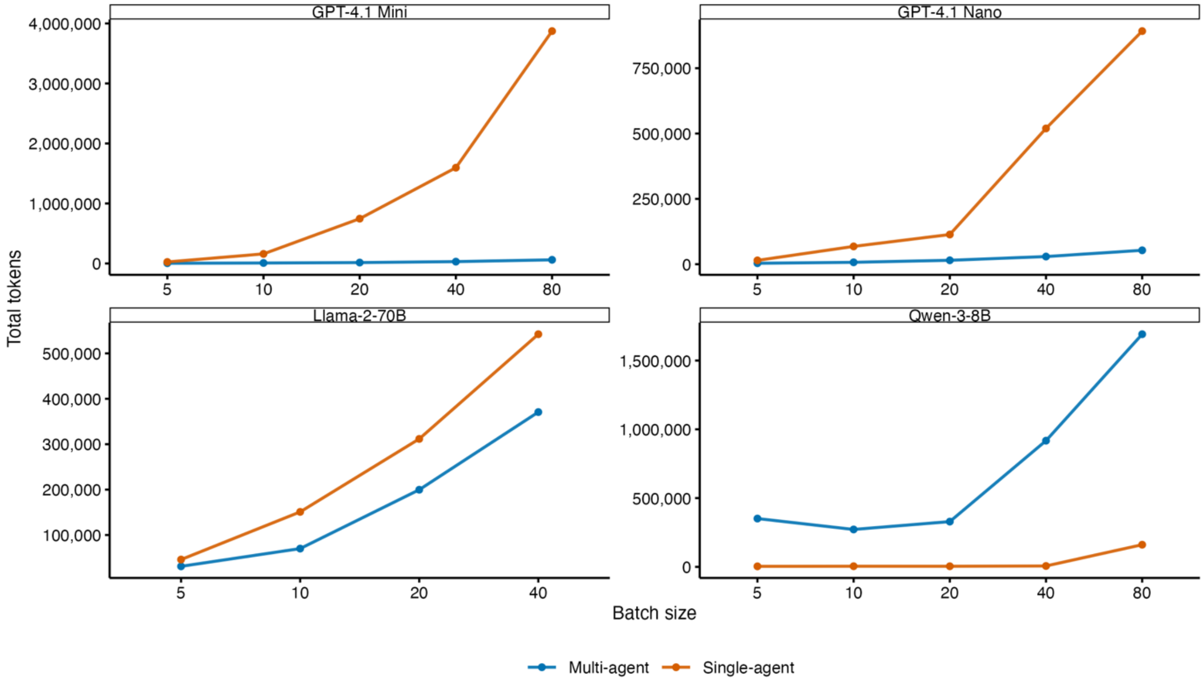

Token utilization patterns across different model architectures. Llama-2-70B data available up to batch size 40.

Figure S3: Token consupmtion by agent type and batch size.

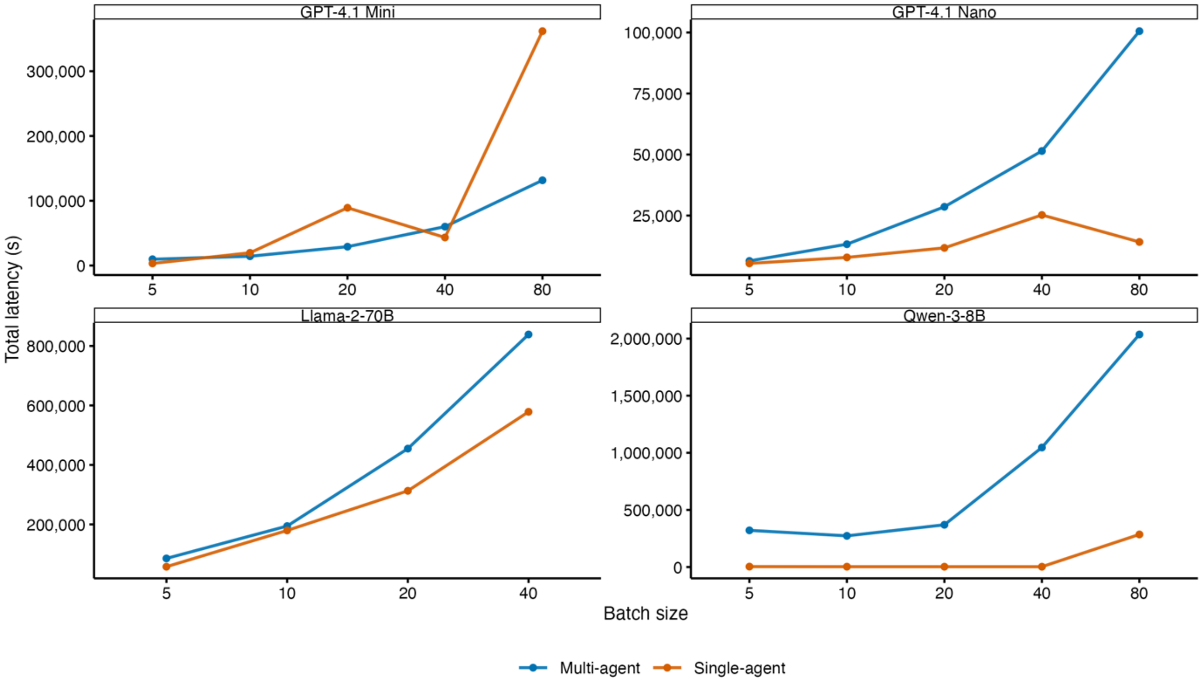

Latency scaling patterns vary across model architectures. Llama-2-70B evaluated up to batch size 40, other models up to 80.

Figure S2: Computational latency by agnet type and batch size.

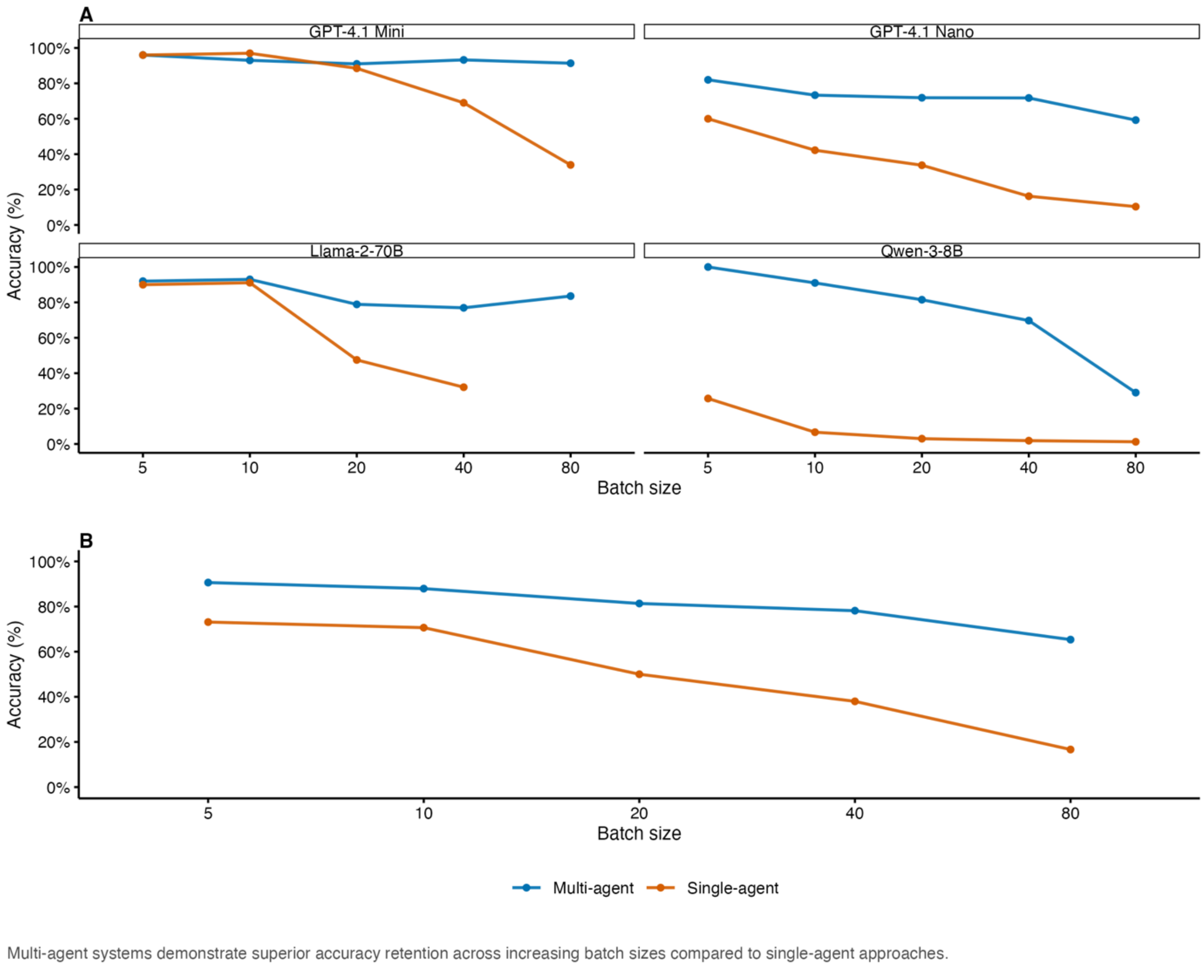

Figure S1: Task accuracy by agent type and batch size.
*Many single-agent runs, especially the larger batches for Llama-2-70B and Qwen-3-8B, terminated early with error JSON. These failures inflate single-agent latency and token counts in the comparison with the multi-agent runs.

* For Qwen-3-8B single-agent, values across N=5–40 vary modestly and can appear visually flat due to axis scaling; the numeric values are provided in Tables S2–S3.

Section 2: Detailed data construction, validation and access details.

1. *Data sources and ground truth*

We used three public corpora.

Literature retrieval. PubMed abstracts tagged *Neoplasms* and dated 2020-01-01 to 2025-05-30 (n = 234 650) were embedded with GIST-large (768 D, L2 normalised) and stored in a FAISS inner-product index.

Clinical-note extraction. All discharge summaries in MIMIC-IV that include the headers *Sex*, *Admission Date* and *Service* (n = 331 793) were retained. Admission dates were resampled uniformly across 2005-01-01 to 2019-12-31; discharge dates were offset 2–14 days later.

Medication maths. Twenty deterministic templates generated dosing questions that cover weight-based, body-surface-area, BMI, Cockcroft–Gault, infusion-rate and concentration conversions. Template parameters were drawn from uniform ranges.

Each task has one canonical answer string. Exact-match accuracy was computed after case-folding and whitespace normalisation.

2. *Tools exposed to agents*

Retriever returns the *k* nearest abstracts plus cosine scores.

Calculator evaluates arithmetic expressions in a sandbox limited to Python's public *math* functions.

Note fetcher returns the full discharge summary for a given identifier.

3. *Agent topologies*

Single agent (SA). One LLM receives the whole batch of *N* tasks and may use up to 10 × *N* dialogue turns.

Multi agent (MA). A lightweight orchestrator issues one *solve_task* call per task, passes the payload to a dedicated worker that may invoke one tool, and aggregates the worker outputs.

Four instruction-tuned checkpoints—GPT-4.1-mini, GPT-4.1-nano, Llama-2-70B-chat and Qwen-3-8B-chat—ran under both topologies. Random seed = 42.

4. *Experimental design*

Batch sizes were 5, 10, 20, 40 and 80. Ten non-overlapping batches were generated for every (model × topology × batch size) cell. Open-weight models ran on one node with 8 × NVIDIA

H100-80 GB GPUs; hosted models used vendor APIs. Wall-clock latency was measured from the first model call to receipt of the final JSON. Total input and output tokens per batch were logged and divided by *N* for per-task values.

5. *Statistical analysis*

Accuracy was compared with two-sided Welch t-tests. Total latency and token counts, which were skewed, used Wilcoxon rank-sum tests. P values were adjusted within each metric family by the Benjamini–Hochberg method. Analyses were performed in R 4.3.

Section 3: Codes for running the agent pipelines.
Orchestrator pipeline

--- Code Cell 1 ---

```
import json, ast, re, difflib, pandas as pd

def calculate_accuracy (result, df_tasks):

    try:

        agent_answers = ast.literal_eval (result)                # {"answer1": "...", ...}

        def clean(txt: object) -> str:
            """Lowercase, strip, drop quotes/commas/spaces."""
            return re.sub(r"[\"',]", "", str(txt)).strip().lower()

        def similar(a: str, b: str, thresh: float = 0.95) -> bool:
            """True if similarity ratio ≥ thresh."""
            ratio = difflib.SequenceMatcher(None, a, b).ratio()
            return ratio >= thresh

        results = []
        for i, gold in enumerate(df_tasks["gold_answer"], start=1):

            pred  = agent_answers.get(f"answer{i}", "")

            if pred.lower () == "male": pred = "M"

            if pred.lower () == "female": pred = "F"

            results.append (similar (clean(gold), clean(pred)))

        accuracy = sum(results) / len(results)

        return accuracy

    except:

        return "FAILED JSON"
```

--- Code Cell 2 ---

```
import pandas as pd
from pandas import NA
import time
```

```
def tag_and_accumulate(
    df: pd.DataFrame,
) -> pd.DataFrame:
    """
    Label every row and build cumulative-context columns, now INCLUDING
    each agent's system prompt.

    Parameters
    ----------
    df : DataFrame with columns agent, role, content
    orch_system : str  (orchestrator's system prompt)
    worker_system : str  (worker system prompt, same for every worker)

    Returns
    -------
    DataFrame  (original + flag cols + orch_cumulative + worker_cumulative)
    """

    orch_system = """
You are an agent.

You will receive N independent clinical tasks.

For each task, call 'solve_task' (task) **one at a time**.

Only after all tasks are solved, return all the answers as:

{"answer1":"...", "answer2":"..." ... "answerN":"..."}

The answers must be just one term, no extra text, explanations or units."""

    worker_system = """
You are an agent.

You will recieve one task.

Choose one tool for this task:

• calculator(expression)
• pubmed_abstracts(query, top_k)
• note_text(note_id)

Return the answer as just one term, no extra text, explanations or units."""
```

```
# ── basic masks
is_orch   = df["agent"] == "orchestrator"
is_worker = df["agent"] == "worker"
is_tool   = df["agent"] == "tool"

# ── worker-level flags
worker_input  = (
    (is_worker & (df["role"] == "user")) |
    (is_tool   & df["role"].str.endswith(".output", na=False))
)

worker_output = (
    (is_worker & (df["role"] == "assistant")) |
    (is_tool   & df["role"].str.endswith(".input",  na=False))
)

# ── orchestrator flags ──────────────────────────────
orch_in  = (
    (is_orch & (df["role"] == "user")) |
    (is_worker & (df["role"] == "assistant"))
)

orch_out = (
    (is_orch & (df["role"] == "assistant")) |
    (is_worker & (df["role"] == "user"))
)

# ── worker index & propagation ──────────────────────
start_flags = is_worker & (df["role"] == "user")
df["worker"] = start_flags.cumsum()
df.loc[~(is_worker | is_tool), "worker"] = NA
df["worker"] = df["worker"].ffill()

# ── attach Boolean columns ──────────────────────────
df["orchestrator input"]  = orch_in.astype("boolean")
df["orchestrator output"] = orch_out.astype("boolean")
df["worker input"]        = worker_input.astype("boolean")
df["worker output"]       = worker_output.astype("boolean")

# ── cumulative context columns ──────────────────────
df["orch_cumulative"]   = NA
df["worker_cumulative"] = NA

# initialise the histories with the system prompts
```

```
    orch_hist: list[str] = [orch_system] if orch_system else []
    worker_hist: dict[int, list[str]] = {}

    for idx, row in df.iterrows():
        content = str(row["content"])

        # ─ orchestrator history grows on every row it sends/receives
        if is_orch[idx] or (is_worker[idx] and not is_tool[idx]):
            orch_hist.append(content)

        # ─ worker history (per-ID) grows on every worker/tool row
        wid = row["worker"]
        if pd.notna(wid):
            wid = int(wid)
            if wid not in worker_hist:
                # first time we meet this worker → start with system prompt
                worker_hist[wid] = [worker_system] if worker_system else []
            worker_hist[wid].append(content)

        # ─ snapshots taken only on *input* rows
        if orch_in[idx]:
            df.at[idx, "orch_cumulative"] = "\n".join(orch_hist)

        if worker_input[idx]:
            df.at[idx, "worker_cumulative"] = "\n".join(worker_hist[int(wid)])

    return df


--- Code Cell 3 ---
import tiktoken

def summarize_token_usage(df):
    """
    Print the total token count in each message category
    (orchestrator input/output, worker input/output).

    Assumes the DataFrame already has Boolean columns:
        • orchestrator input
        • orchestrator output
        • worker input
        • worker output
    and a `content` column holding the text.
    """
    enc = tiktoken.get_encoding("cl100k_base")  # GPT-4 family
```

```
    def token_sum(mask):
        return sum(len(enc.encode(str(text)))
                   for text in df.loc[mask, "content"])

    totals = {
        "orch_in":   token_sum(df["orchestrator input"]),
        "orch_out":  token_sum(df["orchestrator output"]),
        "worker_in": token_sum(df["worker input"]),
        "worker_out":token_sum(df["worker output"]),
    }

    return totals
```

--- Code Cell 4 ---

```
import time
import asyncio
import nest_asyncio
from pathlib import Path
from datetime import datetime
import json
from typing import Any, Dict, List

import pandas as pd
import tiktoken
from openai import AsyncOpenAI
from agents import (
    Agent, Runner, OpenAIChatCompletionsModel, ModelSettings,
    function_tool, set_default_openai_client, set_default_openai_api,
    set_tracing_disabled
)

# ── logging helpers
```

```
encoder = tiktoken.get_encoding("cl100k_base")  # fallback encoding works for GPT-4 variants


def log_message(agent: str, role: str, content: str):
    """Record one message; write everything once, at the very end."""
    _LOG.append({
        "time":   datetime.utcnow().isoformat(timespec="seconds"),
        "agent":  agent,
        "role":   role,
```

```
        "tokens": len(encoder.encode(content)),
        "content": content,
    })


# ── data helpers
```

```
from calculator import Calculator
from pubmed_rag_index import SimplePubmedRAG


def build_openai_client():
    """Initialise the AsyncOpenAI client for Azure deployment."""
    nest_asyncio.apply()
    key_file = Path("azure openai research.txt")
    AZURE_KEY, AZURE_ENDPOINT = key_file.read_text().splitlines()[:2]

    # MODEL_DEPLOYMENT = "gpt-4o-mini-deploy-test-001"
    # MODEL_DEPLOYMENT = "gpt-41-nano-dzs-d3m-sandbox-swc-01-01"
    MODEL_DEPLOYMENT = "gpt-41-mini-dzs-d3m-sandbox-swc-01-01"
    # MODEL_DEPLOYMENT = "gpt-41-dzs-d3m-sandbox-swc-01-01"
    # MODEL_DEPLOYMENT = "gpt-o3-d3m-sandbox-clinicalrounding-swc-01-01"

    API_VERSION = "2024-12-01-preview"

    client = AsyncOpenAI(
        api_key=AZURE_KEY,

base_url=f"{AZURE_ENDPOINT.rstrip('/')}/openai/deployments/{MODEL_DEPLOYMENT}"
,
        default_headers={"api-key": AZURE_KEY},
        default_query={"api-version": API_VERSION},
    )
    set_default_openai_client(client, use_for_tracing=False)
    set_default_openai_api("chat_completions")
    set_tracing_disabled(True)
    return client, MODEL_DEPLOYMENT


client, MODEL_DEPLOYMENT = build_openai_client()

# ── load external data
```

```
df_notes = pd.read_pickle("notes/notes_10000.pkl")
```

```
df_abstracts = pd.read_pickle("pubmed/abstracts.pkl")

calc = Calculator()
rag = SimplePubmedRAG()
rag.load()

# ── domain tools with logging ─────────────────────────────────────


@function_tool
def calculator(expression: str) -> str:
    """Return the numeric result of an arithmetic expression."""
    log_message ("tool", "calculator.input", expression)
    result = f"{calc.calculate(expression)['value']:.2f}"
    log_message ("tool", "calculator.output", result)
    return result


@function_tool
def pubmed_abstracts(query: str, top_k: int = 3) -> dict[str, str]:
    """Return {PMID: abstract_text} for the best matching PubMed hits."""
    log_message("tool", "pubmed_abstracts.input", query)
    hits = rag.search(query, top_k=top_k)
    abstracts = {
        str(h["PMID"]): df_abstracts.loc[
            df_abstracts["PMID"] == h["PMID"]
        ].iloc[0]["abstract"]
        for h in hits
    }
    log_message("tool", "pubmed_abstracts.output", json.dumps(abstracts)[:5000])  # safeguard
gigantic logs
    return abstracts


@function_tool
def note_text(note_id: str) -> str:
    """Fetch full clinical note text by note_id."""
    log_message("tool", "note_text.input", note_id)
    row = df_notes.loc[df_notes["note_id"] == note_id]
    text = "" if row.empty else row.iloc[0]["text"]
    log_message("tool", "note_text.output", text[:5000])
    return text

# ── worker agent ──────────────────────────────────────────────────
```

```
worker_agent = Agent(
    name="Clinical-Worker",
    instructions=(
"""
You are an agent.

You will recieve one task.

Choose one tool for this task:

• calculator(expression)
• pubmed_abstracts(query, top_k)
• note_text(note_id)

Return the answer as just one term, no extra text, explanations or units.
"""
    ),
    model=OpenAIChatCompletionsModel(model=MODEL_DEPLOYMENT,
openai_client=client),
    model_settings=ModelSettings(),
    tools=[calculator, pubmed_abstracts, note_text],
)

# ── expose the worker as a tool, with logging ────────────────────────

def make_worker_tool(agent: Agent):
    @function_tool(
        name_override="solve_task",
        description_override="Solve a single clinical task and return the answer."
    )
    async def solve_task(task: str) -> str:

        log_message("worker", "user", task)

        result = await Runner.run (agent, task, max_turns=10)

        # ── worker's answer (output) ──
        log_message("worker", "assistant", result.final_output)

        return result.final_output

    return solve_task

solve_task = make_worker_tool(worker_agent)
```

```
# ── orchestrator agent

orchestrator_agent = Agent(
    name="Orchestrator",
    instructions=(
"""
You are an agent.

You will receive N independent clinical tasks.

For each task, call 'solve_task' (task) **one at a time**.

Only after all tasks are solved, return all the answers as:

{"answer1":"...", "answer2":"..." ... "answerN":"..."}

The answers must be just one term, no extra text, explanations or units.
"""
    ),
    model=OpenAIChatCompletionsModel(model=MODEL_DEPLOYMENT,
openai_client=client),
    model_settings=ModelSettings(parallel_tool_calls=False),
    tools=[solve_task],
)

# ── helper to run a batch of tasks

def run_batch(tasks: List[str], max_turns: int = 200):

    composite_prompt = (
        "Here are the tasks. Solve them one by one:\n\n" +
        "\n".join(f"{i+1}. {t}" for i, t in enumerate(tasks))
    )

    log_message("orchestrator", "user", composite_prompt)

    result = Runner.run_sync (orchestrator_agent, composite_prompt, max_turns=max_turns)

    # log the orchestrator's reply
    log_message("orchestrator", "assistant", result.final_output)

    return result
```

```
# ── example entry point

df_all = pd.read_pickle("tasks_set.pkl")

_LOG = []

offset = 0                    # running position in df_all

for N_tasks in [5, 10, 20, 40, 80]:

    for exp in range(1, 11):      # 1-based for nicer file names

        _LOG.clear()            # start fresh

        df_tasks = df_all.iloc [offset:offset + N_tasks]

        offset += N_tasks       # advance for next run

        stem = f"multi_{MODEL_DEPLOYMENT}_{N_tasks}_{exp}"

        # Ensure the directory exists; create it if it does not
        Path(f"singles/{MODEL_DEPLOYMENT}").mkdir(parents=True, exist_ok=True)

        log_path = Path(f"singles/{MODEL_DEPLOYMENT}") / f"{stem}_log.pkl"

        if log_path.exists():

            print (f"{log_path} already exists – skipping round")

            continue

        start = time.perf_counter()
        result = run_batch (df_tasks["prompt"].tolist())
        latency_ms = int ((time.perf_counter() - start) * 1000)

        df = pd.DataFrame(_LOG)

        df = tag_and_accumulate (df)

        df.to_pickle(f"singles/{MODEL_DEPLOYMENT}/{stem}_log.pkl")

        df_tasks.to_pickle(f"singles/{MODEL_DEPLOYMENT}/{stem}_tasks.pkl")

        accuracy = calculate_accuracy (result.final_output, df_tasks)
```

```
totals = summarize_token_usage (df)

df_summarize = pd.DataFrame ({
    "offset":[offset],
    "latency":[latency_ms],
    "model":[MODEL_DEPLOYMENT],
    "exp":[exp],
    "N_tasks":[N_tasks],
    "accuracy":[accuracy],
    "input_tokens" : [totals ["orch_in"] + totals ["worker_in"]],
    "output_tokens" : [totals ["orch_out"] + totals ["worker_out"]],
    "orchestrator_input_tokens" : [totals ["orch_in"]],
    "orchestrator_output_tokens" : [totals ["orch_out"]],
    "worker_input_tokens" : [totals ["worker_in"]],
    "worker_output_tokens" : [totals ["worker_out"]]
})

df_summarize.to_pickle (f"singles/{MODEL_DEPLOYMENT}/{stem}_summarize.pkl")

print (stem, latency_ms, accuracy, totals)
```

Singel agent pipeline:

--- Code Cell 1 ---

```
import json, ast, re, difflib, pandas as pd

def calculate_accuracy (result, df_tasks):

    try:

        agent_answers = ast.literal_eval (result)                # {"answer1": "...", ...}

        def clean(txt: object) -> str:
            """Lowercase, strip, drop quotes/commas/spaces."""
            return re.sub(r"[\"',]", "", str(txt)).strip().lower()

        def similar(a: str, b: str, thresh: float = 0.95) -> bool:
            """True if similarity ratio ≥ thresh."""
            ratio = difflib.SequenceMatcher(None, a, b).ratio()
            return ratio >= thresh

        results = []

        for i, gold in enumerate (df_tasks["gold_answer"], start=1):

            pred  = agent_answers.get(f"answer{i}", "")

            if pred.lower () == "male": pred = "M"

            if pred.lower () == "female": pred = "F"

            results.append (similar (clean(gold), clean(pred)))

        accuracy = sum(results) / len(results)

        return accuracy

    except:

        return "FAILED JSON"
```

--- Code Cell 2 ---

```
import pandas as pd
import tiktoken


def enrich_chat_log (df: pd.DataFrame,
                 system_prompt: str = "this is system prompt") -> pd.DataFrame:
   """
   Adds chat-aware token-count columns to a conversation trace.

   New columns
   -----------
   input_tokens  – tokens in the current row that the model receives
   output_tokens – tokens in the current row that the model emits
   chat          – total tokens in the running chat after this row
   real_input    – chat for input rows, 0 for output rows
   """
   enc = tiktoken.get_encoding("cl100k_base")
   sys_tokens = len(enc.encode(system_prompt))

   def n_tokens(txt) -> int:
      return len(enc.encode(str(txt)))

   def classify(row):
      """Return (in_tok, out_tok, is_input_row)."""
      if row.get("role") in ("user", "tool") or row.get("type") == "function_call_output":
         txt = row.get("content") if pd.notnull(row.get("content")) else row.get("output")
         t = n_tokens(txt)
         return t, 0, True
      txt = row.get("content") if pd.notnull(row.get("content")) else row.get("arguments")
      t = n_tokens(txt)
      return 0, t, False


   cols = df.apply(classify, axis=1, result_type="expand")

   cols.columns = ["input_tokens", "output_tokens", "is_input"]

   df = pd.concat([df, cols], axis=1)

   chat_tokens = sys_tokens
   chat_list, real_input_list = [], []

   for in_tok, out_tok, is_in in df[["input_tokens", "output_tokens",
"is_input"]].itertuples(index=False):
      if is_in:
         chat_tokens += in_tok
```

```
            real_input_list.append(chat_tokens)
        else:
            chat_tokens += out_tok
            real_input_list.append(0)
        chat_list.append(chat_tokens)

    df["chat"] = chat_list

    df["real_input"] = real_input_list

    return df
```

--- Code Cell 3 ---

```
"""
Single-agent stress test: clinical agent → three tools
Revised: give the agent many tasks at once; it answers them sequentially
and returns a JSON array of answers.
"""

import time
import nest_asyncio
import pandas as pd
from pathlib import Path
from openai import AsyncOpenAI
from agents import (                        # same SDK import block
    Agent, Runner, OpenAIChatCompletionsModel, ModelSettings,
    function_tool, set_default_openai_client, set_default_openai_api,
    set_tracing_disabled,
    ItemHelpers, MessageOutputItem, ToolCallItem,
    ToolCallOutputItem, ReasoningItem,
)

# ── initial setup identical to your original file ──────────────────────
nest_asyncio.apply()
key_file = Path("azure openai research.txt")
AZURE_KEY, AZURE_ENDPOINT = key_file.read_text().splitlines()[:2]

# MODEL_DEPLOYMENT = "gpt-4o-mini-deploy-test-001"
# MODEL_DEPLOYMENT = "gpt-41-nano-dzs-d3m-sandbox-swc-01-01"
MODEL_DEPLOYMENT = "gpt-41-mini-dzs-d3m-sandbox-swc-01-01"
# MODEL_DEPLOYMENT = "gpt-41-dzs-d3m-sandbox-swc-01-01"

API_VERSION      = "2024-12-01-preview"

client = AsyncOpenAI(
```

```
    api_key=AZURE_KEY,

base_url=f"{AZURE_ENDPOINT.rstrip('/')}/openai/deployments/{MODEL_DEPLOYMENT}"
,
    default_headers={"api-key": AZURE_KEY},
    default_query={"api-version": API_VERSION},
)
set_default_openai_client(client, use_for_tracing=False)
set_default_openai_api("chat_completions")
set_tracing_disabled(True)

# ── external data and tools (unchanged)
────────────────────────────────────────
df_notes     = pd.read_pickle("notes/notes_10000.pkl")
df_abstracts = pd.read_pickle("pubmed/abstracts.pkl")

from calculator import Calculator
from pubmed_rag_index import SimplePubmedRAG
rag = SimplePubmedRAG(); rag.load()
calc = Calculator()

@function_tool
def calculator(expression: str) -> str:
    """Return the numeric result of an arithmetic expression."""
    return str(calc.calculate(expression)["value"])

@function_tool
def pubmed_abstracts(query: str, top_k: int = 3) -> dict[str, str]:
    """Return {PMID: abstract_text} for the best matching PubMed hits."""
    hits = rag.search(query, top_k=top_k)
    return {
        str(h["PMID"]): df_abstracts.loc[
            df_abstracts["PMID"] == h["PMID"]
        ].iloc[0]["abstract"]
        for h in hits
    }

@function_tool
def note_text(note_id: str) -> str:
    """Fetch full clinical note text by note_id."""
    row = df_notes.loc[df_notes["note_id"] == note_id]
    return "" if row.empty else row.iloc[0]["text"]

# ── clinical agent
──────────────────────────────────────────────────────────────────
clinical_agent = Agent(
```

```
    name="Clinical-Agent",
    instructions=(
"""
You are an agent.

You will receive N independent tasks at once.

Solve them sequentially, one by one using the tools:

• calculator(expression)
• pubmed_abstracts(query, top_k)
• note_text(note_id)

When you have all the answers, return in the format:

{"answer1": <"your answer">, "answer2":"<your answer>"...]}

Each answer must be just one term, without extra text, explanations, units, etc.
"""
    ),
    model=OpenAIChatCompletionsModel(model=MODEL_DEPLOYMENT,
openai_client=client),
    model_settings=ModelSettings(),
    tools=[calculator, pubmed_abstracts, note_text],
)


# ── load tasks and build one composite prompt


df_all = pd.read_pickle("tasks_set.pkl")

offset = 0                    # running position in df_all

for N_tasks in [5, 10, 20, 40, 80]:

    for exp in range (1, 11): # range(1, 6):      # 1-based for nicer file names

        df_tasks = df_all.iloc [offset:offset + N_tasks]

        offset += N_tasks        # advance for next run

        stem = f"single_{MODEL_DEPLOYMENT}_{N_tasks}_{exp}"

        # Ensure the directory exists; create it if it does not
```

```
Path(f"singles/{MODEL_DEPLOYMENT}").mkdir(parents=True, exist_ok=True)

log_path = Path(f"singles/{MODEL_DEPLOYMENT}") / f"{stem}_log.pkl"

if log_path.exists():

    print (f"{log_path} already exists – skipping round")

    continue

multi_task_prompt = (
    "Here are the tasks. Solve them one by one:\n\n" +
    "\n".join(f"{i+1}. {t}" for i, t in enumerate(df_tasks["prompt"].tolist()))
)

t0      = time.monotonic()

result   = Runner.run_sync (clinical_agent, multi_task_prompt,
                        max_turns = 10 * N_tasks)

msgs = result.to_input_list()          # list of plain dicts

df_log = pd.json_normalize(msgs)

df_log = enrich_chat_log (df_log)

latency  = int((time.monotonic() - t0) * 1000)


df_log.to_pickle(f"singles/{MODEL_DEPLOYMENT}/{stem}_log.pkl")

df_tasks.to_pickle(f"singles/{MODEL_DEPLOYMENT}/{stem}_tasks.pkl")

accuracy = calculate_accuracy (result.final_output, df_tasks)

df_summarize = pd.DataFrame ({
    "offset":[offset],
    "latency":[latency],
    "model":[MODEL_DEPLOYMENT],
    "exp":[exp],
    "N_tasks":[N_tasks],
    "accuracy":[accuracy],
    "input_tokens" : [df_log ["real_input"].sum ()],
    "output_tokens" : [df_log ["output_tokens"].sum ()],
})
```

```
df_summarize.to_pickle (f"singles/{MODEL_DEPLOYMENT}/{stem}_summarize.pkl")

print (stem, latency, accuracy,
    df_log ["real_input"].sum (),
    df_log ["output_tokens"].sum ())
```

Section 4: Illustrative task examples

Example 1 — Retrieval (PubMed)

Input (task): “Retrieve the PubMed abstract most relevant to: ‘adjuvant pembrolizumab melanoma trial recurrence-free survival’.”

Expected structured output (JSON): { "retrieved_pmid": "<PMID>", "rank": 1 }

Evaluation: Exact match on retrieved_pmid after normalization.

Example 2 — Extraction (discharge note fields)

Input (note excerpt): “Sex: F … Admission Date: 2016-05-02 … Service: Cardiology …”

Task: “Extract Sex, Admission Date, and Service.”

Expected structured output (JSON): { "sex": "F", "admission_date": "2016-05-02", "service": "Cardiology" }

Evaluation: Exact match after case-folding and whitespace normalization.

Example 3 — Dosing (deterministic calculation)

Input: “Patient weight is 70 kg. Order is 5 mg/kg/day divided into 2 doses. What is mg per dose?”

Expected structured output (JSON): { "mg_per_dose": 175 }

Evaluation: Exact match after normalization.
